# Experiences of psychological, social and organisational work environments in occupational health service in Sweden: a cross-sectional survey

**DOI:** 10.1186/s12913-024-11766-7

**Published:** 2024-10-29

**Authors:** Anna-Karin Mouazzen, Karin Blomberg, Maria Jaensson

**Affiliations:** School of Health Sciences, Faculty of Medicine and Health, Örebro University, Fakultetsgatan 1, Örebro, SE-701 82 Sweden

**Keywords:** Occupational health, Occupational health service, Organisational and social factors at work, Psychosocial working conditions, QPS_Nordic_

## Abstract

**Background:**

The aim of this study was to compare and describe different professionals’ experiences of workplace psychological and social factors in occupational health (OH) organizations in Sweden.

**Methods:**

This cross-sectional study with a descriptive and comparative design included 472 respondents with common professions in the occupational health service (OHS) in Sweden. Data were collected with “The General Nordic Questionnaire for Psychological and Social Factors at Work” (QPS_Nordic)_. The professions have been compared pairwise using the Independent Samples Kruskal-Wallis test, adjusted by Bonferroni correction for multiple tests on subscales and single items and these are presented descriptively.

**Results:**

The experience of the psychological and social work environment on job task measurement level differed between the professionals. Experiences on social and organizational as well as on individual measurement levels are similar between the professionals who perceive them as satisfactory. Out of the 472 respondents, 7% reported that they had seen someone being subjected to harassment and bullying at the workplace during the last six months.

**Conclusions:**

The experience among the professionals differs most in the Job task measurement level. The results indicate that although different OH professionals experience psychological and social factors at work in different ways, their experiences are generally satisfactory even though harassment and bullying do exist. The research about occupational health professionals and their work environment is sparse. Further applied research is needed for the planning and development of occupational health services in Sweden.

## Background

Psychological, social and organisational factors at work, are complex phenomena which affect the health and well-being of the working population [1, 2]. Within this sphere of complex phenomena are different occurrences, circumstances and conditions that require the individual to respond to and act upon at the workplace [1]. This can be exemplified as role expectations, mastery of work, social interactions, leadership and the organizational culture and climate [3]. All these aspects contribute towards complex workplace phenomena and they could also be characterised as demands of the job, victimisation, unhealthy workload, organisational environment, resources for the job and social work environment [2]. With suboptimal psychological, social and organisational working conditions there are risks for mental health problems, physical health problems and sickness absence in the workplace [1, 4]. When there is low social support, long working hours, job strain, organisational injustice, low decision latitude, job insecurity, bullying or other unhealthy exposures at work a person is at risk for developing cardiovascular diseases, depression, burnout, increased alcohol intake, sleeping problems and/or suicidal ideations [1]. When addressing subjects of psychological, social and organizational work environment in research, “Psychosocial work environment” is the most frequently used term [5]. The focus has then been on the individual level rather than the on organizational level [1, 5, 6]. Therefore, a more meaningful and correct term to use is “psychological, social and organisational work environment” to be able to work systematic with the work environment within organizations [5]. The work load and the work-related health may also be influenced by the physical and ergonomic conditions in the workplace [2].

A systematic approach is helpful when developing healthy workplaces. The work environment must be analysed. This means assessing the existing organizational environment and taking measures to reduce any risks and then checking whether corrective measures have contributed to a better work environment [2, 7]. In Sweden it is the employer`s obligation, regulated by the so-called “Systematic work environment management” to develop the work environment [7]. Any employer with more than ten employees is obliged to have a provider of occupational health (OH) available, in accordance with the required working conditions [8]. Occupational health service (OHS) refers to as an independent resource which has expert knowledge about work environments and rehabilitation. The OH provider should have the competence to identify and describe the relation between work environments, organization, productivity and health, and to suggest actions for improving the work environment when necessary [8].

The professionals working within the OHS are specialists in occupational safety and health and/or occupational medicine. Their role is to investigate, carry out risk assessments to identify occupational exposures and to give advice for remedying and evaluating remedial efforts that have been undertaken together with their customers i.e., employers and organizations [9, 10]. They use their evidence-based knowledge when working with developing and improving others´ work environments. To be able to develop the work environment in OHS an increased knowledge of the psychological, social and organizational work environment is needed for developing enhanced workplace strategies by OHS professionals with and for the clients they support. Therefore, the aim of this study was to describe and compare different professionals’ experiences of psychological and social factors at the workplace in OH organizations in Sweden.

## Method

### Study design

A descriptive comparative design was used for the study.

### Setting

Occupational health services in Sweden are organized as in-house OH services within private or public organizations for their own employees, or as a private OH having agreements to offer/sell their services to other organizations. The term Occupational health service (OHS) is usually used to describe the type of business (cf. healthcare) and the provider of occupational health care is usually referred to as Occupational Health (OH) (cf. health care providers). The OHS and the activities conducted within the setting are not mandated by any governmental authority or other public organization. To be a member of the Swedish trade association of occupational health service “Sveriges Företagshälsor” the OHS needs to have certain educational requirements. Organizations and their units which met such requirements were eligible for inclusion in this study.

### Sample

A convenience sample was used. The eligible participants (*n* = 2,035) who were invited comprised different professional groups (i.e., behavioural scientists/psychologists, ergonomists/physiotherapists, physicians, registered nurses and safety and health engineers) working at OH units in Sweden. At the time of the study 407 units in Sweden were registered with the trade association.

### Data collection

The data collection took place from October 2018 to June 2019. Managers at OH units received a letter containing information about the study and were asked to choose five co-workers with different professions who would be willing to complete a survey. Obtaining personal addresses to the professionals within the OHS is not possible due to the General Data Protection Regulation (GDPR) therefore the survey was sent out for distribution by the manager approximately 2 weeks later. A letter was attached with information about the survey and with the assurance that participation in the study was voluntary. Due to invalid addresses some questionnaires were returned unopened (*n* = 112). Some OH organizations, units and some individual professionals declined participation for reasons of upcoming reorganizations and lack of time. Some replied that they had received two surveys as they were working at more than one unit, and that they would only answer one survey. No reminder letters were sent out.

The survey included two instruments, the General Nordic Questionnaire for Psychological and Social Factors at work (QPS_Nordic_) [11], the Assessment of Interprofessional Team Collaboration Scale adapted for OH (AITCS-S(OH)) [12] and an open-ended question. Data from the shortened version of AITCS-S(OH), AITCS-SII(OH) and the open-ended-question have been reported elsewhere [12, 13].

### Instrument

QPS_Nordic_ is a complex instrument which measures psychological and social factors at work in a comprehensive manner on different levels without any hierarchical order [14].

The original QPS_Nordic_ contains 129 questions, 11 background questions and 118 further questions. In the present study some of the background questions were modified for the current context. In addition to questions about participants’ age, sex and profession, information was also obtained about professionals’ education in safety and health, their type of employment (full time/consultant), their working hours and years of work experience and their length of employment at their current workplace. Also noted was the nature of the company - whether it was service or industrial and if it was a private/in house/public organisation and the number of inhabitants in the municipality. Out of the 118 questions 80 questions are distributed in 26 subscales and 38 are single items. QPS Nordic also incorporates content areas and different measurement levels (see Table 1) [15]. Most questions are rated on a 5-point Likert scale with the alternatives expressed in ways appropriate to the question, for example “very seldom or never/very low or not at all”, “rather seldom/fairly little”, “sometimes/some”, “rather often/pretty much” and “very often or always/very much”. There are three questions with the option to answer yes or no, and if answering yes there are further supplementary questions. The subscales and/or content areas “*Work centrality”*, “*Interaction between work and private life*” and “*Support from friends and relatives*” are not reported in this study, since they do not refer to job-based psychological and social factors. The demographic variables: working hour/week and number of inhabitants in the municipality where the OH was placed were also excluded since they were assumed to give no further information about the professional’s experience of psychological and social factors at work.

**Table 1:** The structure of QPSNordic with the measurement levels, content areas and subscales

Job task^a^	Social and organizational^a^	Individual^a^
***Job demands*** ^***b***^ -quantitative demands^c^ -decision demands^c^ -learning demands^c^ ***Role expectations*** ^***b***^ -role clarity^c^ -role conflict^c^ ***Control at work*** ^***b***^ -positive challenges at work^c^ -control of decision^c^ -control of work pacing^c^ ***Predictability at work***^b^* -predictability during the next month^c^	***Social interactions*** ^***b***^ -support from superior^c^ -support from coworkers^c^ -support from friends and relatives^c^ - harassment and bullying^c^ ***Leadership*** ^***b***^ -empowering leadership^c^ -fair leadership^c^ ***Organizational culture and climate*** ^***b***^ -social climate^c^ -innovative climate^c^ -inequality^c^ -human resource primacy^c^ ***Perception of groupwork*** ^***b+c***^	***Predictability at work***^b^* -predictability of next two years^c^ -preference for challenge^c^ ***Mastery of work*** ^***b***^ -perception of mastery^c^ ***Interaction between work and private life*** ^***b***^ ***Work centrality*** ^***b+c***^ ***Commitment to organization*** ^***b+c***^ ***Work motives*** ^***b***^ -intrinsic motivation to work^c^ -extrinsic motivation to work^c^

^a^ Measurement levels(*n* = 3)

^b^ Content areas(*n* = 13)

^c^ Subscales (*n* = 26)

^b+c^*Perception of groupwork* and *Commitment to organization* are considered as both content areas and subscales

*Predictability at work is measured both on task level and individual level

The instrument has been tested with different professional groups and different workplaces e.g., in industrial and health care settings [16, 17]. The QPS_Nordic_ has also been psychometrically evaluated and been found both valid and reliable. Internal consistency was evaluated for each measurement level with Cronbach’s alpha between 0.53 and 0.88 for the questions [18]. Reliability was generally satisfactory and confirmatory factor analysis (CFA) indicated good fit when 24 of the 26 scales were included [18].

### Statistical analysis

Demographic variables were presented as number (n), percentage (%), mean, standard deviation (SD), median and range. To show the nature of missing data, Little´s Missing completely at random (MCAR) test was conducted [19]. To evaluate the internal consistency Cronbach’s alpha and inter-item correlation were calculated for all reported subscales. A value between 0.70 and 0.90 is a well-accepted guideline for Cronbach’s alpha and inter-item correlations should be between 0.2 and 0.5 [20]. Analyses were made on the original scale with answers from 1 to 5 and on answers from 1 to 3 (1 and 2 were merged together as well as 4 and 5) [21]. Some of the questions are reversed according to the user’s manual [11, 21]. The result presented is the analysis from the merged answers. Mean, (SD), n and % are calculated for response rate, all items and subscales. The professions were compared pairwise with and Independent Samples Kruskal-Wallis test adjusted by Bonferroni correction for multiple tests. All significance tests were two-sided and conducted at a significance level of 5%. Statistical calculations were conducted using Statistical Package of Social Sciences (SPSS) version 26 (IBM Corp., Armonk, NY, USA).

### Ethical considerations

The Swedish Ethical Review Authority, before January 2019 named the Regional Ethical Review Board in Uppsala, gave ethical approval for this study (2018/180). Since there was no possibility to match the answers to the participants, or to a specific OH, the participants were anonymous, and they gave their informed consent by answering and returning the anonymous survey.

## Results

Of 1923 surveys posted, 24% (*n* = 472) were returned. No question in QPS_Nordic_ had more than 3% (*n* = 16) missing answers. Little’s MCAR test showed that all missing data for the demographic variables as well as for QPS_Nordic_ were MCAR, therefore no missing values were replaced.

In Table 2 the demographic data for all respondents are presented. The respondents had a median age of 53 years and a median of 10 years’ experience in OH. Of the 472 respondents 67% (*n* = 314/472) were women. The respondents’ professions were distributed as follows: behavioural scientists or psychologists 20% (*n* = 93/472), ergonomists or physiotherapists 19% (*n* = 89/472), physicians 18% (*n* = 87/472), registered nurses 30% (*n* = 142/472), safety and health engineers 11% (*n* = 54/472) others that included managers or those who had supportive functions 2% (*n* = 7/472). There were few members in the group named “others”, and those in this category were heterogeneous. They are not included in the comparison between professionals. The education in occupational safety and health differed between and within the professions.

**Table 2:** Demographic characteristics of participants (*n* = 472)

	Total	Behavioural scientists/ Psychologists	Ergonomists/ Physio therapists	Physicians	Registered nurses	Safety and health engineers	Others*
n % 472 (100)		93 (20)	89 (19)	87 (18)	142 (30)	54 (11)	7 (2)
***Sex***,*** n (%)***							
Men	151 (32)	27 (29)	29 (33)	52 (60)	8 (6)	34 (63)	1 (14)
Women	314 (67)	65 (70)	59 (66)	35 (40)	129 (91)	20 (37)	6 (86)
Missing	7 (1)	1 (1)	1 (1)	-	5 (3)	-	-
***Age***,*** years***							
Mean (SD)	51.66 (10.49)	48.34 (11.29)	49.54 (10.52)	57.78 (8.49)	50.98 (9.53)	53.77 (9.98)	44.14 (11.02)
Median (range)	53 (25–77)	48 (26–67)	50 (29–77)	60 (36–74)	52 (28–66)	56 (25–69)	47 (29–59)
Missing n (%)	22 (5)	4 (4)	-	4 (4)	9 (6)	1 (2)	-
***Years of experience in OH***							
Mean (SD)	11.42 (9.55)	7.42 (7.47)	13.16 (9.35)	10.74 (7.95)	12.14 (9.63)	14.91 (12.76)	8.71 (7.7)
Median (range)	10 (0–38)	5 (0–32)	11 (0–33)	10 (1–38)	10.50 (0–38)	11.50 (0–38)	8 (1–18)
Missing n (%)	1 (< 1)	1 (< 1)	-	-	-	-	-
***Years working at current Workplace***							
Mean (SD)	6.86 (6.612)	5.38 (5.68)	8.69 (7.62)	6.3 (5.27)	6.71 (6.59)	7.58 (7.71)	8 (7.31)
Median (range)	5 (0–38)	3 (0–30)	6 (0–32)	5 (1–34)	5 (0–38)	4 (0–34)	6 (1–18)
Missing n (%)	33 (7)	6 (6)	4 (4)	4 (4)	13 (9)	4 (7)	2 (3)
***Employment***,*** n (%)***							
Permanently employed	426 (90)	89 (96)	84 (94)	61 (70)	137 (96)	49 (91)	6 (86)
Consultant/Other	41 (7)	4 (4)	4 (4)	24 (28)	3 (2)	5 (9)	1 (14)
Missing n (%)	5 (1)	-	1 (1)	2 (2)	2 (1)	-	-
***Education in OS&H***,*** n (%)***							
Yes	307 (65)	16 (17)	77(86)	66 (95)	95 (67)	54 (100)	-
No	158 (33.5)	75 (81)	11 (12)	17 (20)	47 (33)	-	7 (100)
Missing	7 (1.5)	2 (2)	1 (1)	4 (5)	-	-	-
***Occupational Health***,*** n (%)***							
Private OH nationwide	227 (48)	50 (54)	51 (57)	35 (40)	63 (44)	25 (63)	3 (43)
Private OH with office in more than one place without being nationwide	88 (19)	11 (12)	14 (16)	20 (23)	30 (21)	11 (20)	2 (29)
Private OH with office in one place	31 (6)	5 (5)	5 (6)	6 (7)	10 (7)	3 (6)	2 (29)
In-house OH in a private organization	21 (4)	3 (3)	4 (4)	4 (5)	8 (6)	2 (4)	-
In-house OH in a public organization	83 (18)	18 (19)	15 (17)	14 (16)	26 (18)	11 (20)	-
Missing	22 (5)	6 (6)	-	9 (10)	5 (4)	9 (10)	-
***Type of costumer company***,*** n (%)***							
Service company	82 (17)	19 (20)	11 (12)	26 (30)	22 (16)	4 (7)	-
Industry company	43 (19)	5 (5)	10 (11)	4 (5)	13 (9.2)	11 (20)	-
Mixed	330 (70)	66 (71)	84 (94)	56 (64)	102 (72)	36 (67)	7 (100)
Missing	17 (4)	3 (3)	5 (6)	1 (1)	5 (4)	3 (6)	-

*Abbreviations*: *OS & H* Occupational safety and health, *SD* Standard deviation

*operations manager, naprapath, wellness educator and assistant nurse

### Experience of psychological and social factors at work

In 13 of the 24 subscales and 13 of the 28 single items, there were significant differences between professionals (see Tables 3, 4 and 5). There were more significant differences between professionals in subscales and single items in the measurement level “*Job task”* (Table 3) than in the other measurement levels “*Social and organizational*” (Table 4) and “*Individual”* (Table 5).

### Difference between professionals on the measurement level “job task”

There were significant differences between professionals in *n* = 8/*n* = 9 subscales and *n* = 9/*n* = 14 single items on the measurement level “*Job task”* (see Table 3) but no clear pattern between the professionals was apparent. Registered nurses and physicians reported that error in their work was associated with personal injury and as in the case of safety and health engineers, such error in their work also was associated with a risk of economic losses compared with other professionals (Table 3). Registered nurses reported higher quantitative demands compared with physicians and higher role conflict compared with ergonomists/physiotherapists, physicians and safety and health engineers (Table 3).

**Table 3:** Job task measurement level

Content area, subscales and single items (Number of items included in the subscale) mean (SD)	Behavioural scientists/ Psychologists	Ergonomists/ Physiotherapist	Physicians	Registered nurses	Safety and health engineers	Adjusted *P*-values
***Job demands***						
Quantitative demands (4) a	7.7 (2.07)	7.6 (2.38)	7.0 (2.40)	8.1 (2.39)	7.8 (2.15)	0.004 (Ph/RN)
Decision demands (3) a	7.5 (1.14)	7.2 (1.47)	7.4 (1.49)	7.1 (1.41)	6.6 (1.49)	0.004 (S&H/ Be&Ps) 0.005 (S&H/Ph)
Learning demands (3) a	5.6 (1.33)	5.5 (1.28)	5.0 (4.40)	5.5 (1.15)	5.7 (1.18)	0.022 (Ph/S&H) 0.028 (Ph/Be&Ps) 0.020 (Ph/RN)
Does your work require physical endurance? a	1.3 (0.57)	1.6 (0.76)	1.4 (0.67)	1.5 (0.71)	1.4 (0.66)	NS
Does your work require great precisions of movement? a	1.1 (0.32)	1.3 (0.58)	1.1 (0.32)	1.6 (0.75)	1.2 (0.51)	0.001 (S&H/RN) 0.001 (Er&Psy/RN) 0.000 (Be&Phy/RN)
Are there interruptions that disturb your work? a	1.6 (0.77)	2.1 (0.82)	1.8 (0.32)	2.4 (0.73)	2.2 (0.73)	0.001 (Be&Ps/Er&Phy) 0.001 (Be&Ps/S&H) 0.000 (Be&Ps/RN) 0.000 (Ph/RN)
Is your work monotonous? a	1.2 (0.43)	1.1 (0.34)	1.3 (0.57)	1.3 (0.52)	1.1 (0.27)	0.019 (S&H/RN) 0.012 (S&H/ Ph)
Do you have to repeat the same work procedure at intervals of a few minutes? a	1.2 (0.57)	1.0 (0.21)	1.2 (0.53)	1.2 (0.48)	1.1 (0.32)	0.024 (Er&Phy/RN) 0.045 (Er&Phy/Ph)
Is it possible to have social contacts with coworkers while you are working? c	2.4 (0.71)	2.6 (0.64)	2.5 (0.70)	2.6 (0.63)	2.6 (0.66)	NS
Have you been exposed to threats or violence at work during the last two years? a	1.2 (0.15)	1.0 (0.11)	1.0 (0.11)	1.0 (0.14)	1.0 (0.00)	NS
Are errors in your work associated with a risk of personal injury? a	1.2 (0.53)	1.3 (0.58)	1.6 (0.79)	1.6 (0.72)	1.4 (0.6)	0.033 (Be&Ps/Ph) 0.000 (Be&Ps/RN) 0.011 (Er&Phy/RN)
Are errors in your work associated with a risk of economic losses? a	1.3 (0.59)	1.4 (0.62)	1.7 (0.74)	1.6 (0.72)	1.7 (0.79)	0.002 (Be&Ps/RN) 0.006 (Be&Ps/Ph) 0.006 (Be&Ps/S&H)
Does your work include contacts with customers or clients? c *	3.7 (0.46)	3.8 (0.41)	3.7 (0.49)	3.8 (0.38)	3.9 (0.34)	0.025 (Be&Ps/RN)
Does your work involve personal contacts with customers or clients? c	3.0 (0.18)	3.0 (0.00)	3.0 (0.15)	3.0 (0.145)	2.9 (0.29)	0.013 (S&H/Er&Phy)
Do you have to receive and handle complaints from customer or clients? a	1.5 (0.58)	1.5 (0.59)	1.5 (0.57)	1.8 (0.64)	1.3 (0.47)	0.000 (S&H/RN) 0.001 (Be&Ps/RN) 0.004 (Er&Phy/RN) 0.017 (Ph/RN)
Are you content with your ability to maintain a good relationship with your customers or clients? a	3.0 (0.15)	3.0 (0.15)	2.9 (0.24)	3.0 (0.20)	3.0 (0.19)	NS
***Role expectations***						
Role clarity (3) b	7.9 (1.57)	8.2 (1.33)	8.6 (0.90)	8.2 (1.36)	8.0 (1.37)	0.006 (Be&Ps/Ph) 0.026 (S&H/Ph)
Role conflict (3) a	4.4 (1.47)	4.3 (1.35)	4.4 (1.50)	4.9 (1.53)	4.3 (1.58)	0.020 (Er&Phy/RN) 0.033 (S&H/RN) 0.039 (Ph/RN)
Does your job involve tasks that are in conflict with your personal values? a	1.3 (0.55)	1.1 (0.41)	1.2 (0.48)	1.2 (0.48)	1.1 (0.40)	NS
***Control at work***						
Positive challenge at work (3) b	8.8 (0.48)	8.7 (0.69)	8.7 (0.69)	8.7 (0.70)	8.5 (0.87)	NS
Control of decision (5) b	12.7 (2.11)	11.8 (2.17)	11.4 (2.44)	11.2 (2.40)	12.6 (2.02)	0.002 (RN/S&H) 0.012 (RN/Be&Ps) 0.050 (Ph/S&H)
Control of work pacing (4) b	9.5 (2.38)	10.2 (1.78)	8.9 (2.38)	9.0 (2.30)	11.1 (1.32)	0.005 (Ph/Er&Phy) 0.000 (Ph/S&H) 0.004 (RN/Er&Phy) 0.000 (RN/S&H) 0.000 (Be&Ps/S&H)
***Predictability at work***						
Predictability during the next month (3) b	8.2 (0.98)	7.7 (1.14)	8.7 (1.21)	8.2 (0.99)	7.3 (1.08)	0.000 (S&H/Be&Ps) 0.000(S&H/Ph) 0.000 (S&H/RN) 0.000(Er&Phy/Ph) 0.004 (Er&Phy/RN) 0.026 (ER&Phy/Be&Ps)

*Abbreviations*: *NS* not significant, *Be&Ps* Behavioural scientists/Psychologists, *Er&Psy* Ergonomists/Physiotherapist, *Ph* Physicians, *RN* Registered nurses, *S&H* Safety and health engineers

a = a higher score relates to a negative impact, b = a higher score relates to a positive impact, c = a higher score relates to more contact with coworkers, customers or clients

* Response alternative is 1–4

Analysed with mean (SD) and compared pairwise with Independent Samples Kruskal-Wallis adjusted by Bonferroni correction for multiple tests. Significant at *p* < 0.05

Registered nurses also reported that they received and handled complaints from customers and clients to a greater extent than other professionals (Table 3). Ergonomists/physiotherapists together with safety and health engineers reported a higher control of work pacing compared with other professionals (Table 3). Behavioural scientists/psychologists and physicians reported fewer interruptions that disturb their work compared with the other professionals (Table 3). Physicians reported higher role clarity than behavioural scientists/psychologists and safety and health engineers (Table 3).

### Differences and similarities among professionals on the measurement levels “social and organizational” and “Individual”

On the “*Social and organizational”* measurement level similarities were evident among the professionals. There were significant differences in n = 1/n = 9 subscales and in n = 2/n = 7 single items (see Table 4).

**Table 4:** Social and organizational measurement level

Content area, subscales and single items (Number of items included in the subscale) Mean (SD)	Behavioural scientists/ Psychologists	Ergonomists/ Physiotherapist	Physicians	Registered nurses	Safety and health engineers	Adjusted *P*-values
***Social interactions***						
Support from superior (3) b	7.8 (1.84)	7.7 (1.71)	7.7 (1.74)	7.7 (1.66)	7.9 (1.56)	NS
Support from coworkers (2) b	5.6 (0.82)	5.4 (1.08)	5.5 (0.89)	5.7 (0.77)	5.3 (1.06)	NS
Have you noticed any disturbing conflicts between coworkers? a	1.6 (0.73)	1.5 (0.68)	1.5 (6.8)	1.5 (0.68)	1.4 (0.63)	NS
***Leadership***						
Empowering leadership (3) b	6.9 (2.15)	6.6 (2.07)	6.7 (2.21)	7.11 (2.01)	7.0 (2.01)	NS
Fair leadership (3) b	8.1 (1.45)	8.3 (1.37)	8.2 (1.42)	7.9 (1.57)	8.5 (0.99)	0.041 (RN/S&H)
Do your immediate superior tackle problems as soon as they surface? b	2.5 (0.72)	2.5 (0.75)	2.5 (0.74)	2.4 (0.76)	2.5 (0.77)	NS
Do you trust the ability of the management to look after the future of the company/organization? b	2.6 (0.72)	2.3 (0.77)	2.4 (0.80)	2.4 (0.74)	2.5 (0.73)	NS
***Organizational culture and climate***						
Social climate (3) b	8.2 (1.29)	8.1 (1.42)	7.9 (1.53)	7.9 (1.58)	8.3 (1.10)	NS
Innovative climate (3) b	8.0 (1.37)	8.1 (1.34)	7.7 (1.58)	8.1 (1.32)	8.4 (0.92)	NS
Inequality (2) a	2.4 (0.97)	2.2 (0.61)	2.2 (0.67)	2.4 (0.82)	2.1 (0.37)	NS
Human resource primacy (3)	6.8 (1.82)	6.6 (1.80)	6.7 (2.11)	6.7 (1.97)	7.0 (1.66)	NS
Competitive? ^a^	1.6 (0.78)	2.0 (0.86)	1.7 (0.88)	2.0 (0.84)	1.5 (0.74)	0.047 (S&H/Er&Phy) 0.002 (S&H/RN) 0.053 (Be&Ps/Er&Phy) 0.001 (Be&Ps/RN) 0.015 (Ph/RN)
Rigid and rule-based?	1.4 (0.65)	1.4 (0.66)	1.5 (0.76)	1.5 (0.69)	1.4 (0.63)	NS
***Perception of group work*** (3)	8.1 (1.40)	8.5 (1.00)	8.3 (1.35)	8.4 (1.15)	8.6 (0.81)	NS
Do you belong to a permanent working group or team? responding yes n (%)	72 (77)	61 (68)	69 (79)	116 (82)	23 (24)	0.010 (S&H/Er&Psy) 0.000 (S&H/Be&Ps) 0.000 (S&H/Ph) 0.000 (S&H/RN)
How often does your group or team have group or team meetings? **	2.5 (0.71)	2.6 (0.69)	2.7 (0.60)	2.6 (0.60)	2.7 (0.60)	NS

*Abbreviations*: *NS* not significant, *Be&Ps* Behavioural scientists/Psychologists, *Er&Psy* Ergonomists/Physiotherapist, *Ph* Physicians, *RN* Registered nurses, *S&H* Safety and health engineers

a = a higher score relates to a negative impact, b = a higher score relates to a positive impact. **The response rate is calculated on 353 respondents, those answering yes and missing on item number 112. Analysed with mean (SD) and compared pairwise with Independent Samples Kruskal-Wallis adjusted by Bonferroni correction for multiple tests

Significant at *p* < 0.05

There were significant differences between the professionals in the *n* = 4/*n* = 6 subscales and significant differences in *n* = 2/*n* = 7 single items on “*Individual”* measurement level (see Table 5).

**Table 5:** Individual measurement level

Content area, subscales and single items (Number of items included in the scales) Mean (SD)	Behavioural scientists/ Psychologists	Ergonomists/ Physiotherapist	Physicians	Registered nurses	Safety and health engineers	Adjusted *P*-values
***Predictability at work***						
Predictability of next two years (2) b	4.8 (1.56)	4.3 (1.71)	5.3 (1.27)	4.6 (1.62)	4.6 (1.63)	0.000 (Er&Phy/Ph) 0.043 (S&H/Ph) 0.004 (RN/Ph)
Preference for challenge (3) b	6.7 (1.79)	7.5 (1.55)	6.9 (1.61)	7.3 (1.45)	8.0 (1.13)	0.011 (Be&Ps/Er&Phy) 0.000 (Be&Ps/S&Hb) 0.000 (Ph/S&H) 0.041 (RN/S&H)
Is it necessary to demonstrate your ability and competence to others in order to be assigned to attractive tasks or projects? a	1.6 (0.73)	1.6 (0.76)	1.3 (0.58)	1.6 (0.74)	1.5 (0.75)	0.030 (Ph/Er&Phy) 0.004 (Ph/RN) 0.007 (Ph/Be&Ps)
Do you feel that you have someone or an organization who looks after your interests? b	2.0 (0.88)	1.9 (0.91)	1.9 (0.91)	2.0 (0.82)	2.5 (0.74)	NS
Are there rumours concerning changes at your workplace? a	1.7 (0.74)	1.6 (0.71)	1.6 (0.78)	1.7 (0.79)	1.4 (0.63)	NS
Are you confident that in 2 years from now you will have a job that you consider as attractive as your present job? b	2.8 (0.53)	2.6 (0.69)	2.8 (0.49)	2.7 (0.63)	1.4 (0.50)	NS
***Mastery at work***						
Perception of mastery (4) b	11.6 (0.79)	11.6 (0.93)	11.8 (0.66)	11.6 (0.82)	11.4 (1.12)	NS
Do you get information about the quality of work that you do? b	2.2 (0.82)	2.1 (0.74)	2.2 (0.73)	2.2 (0.76)	2.0 (0.78)	NS
Can you yourself immediately assess whether you did your work well? b	2.7 (0.57)	2.7 (0.47)	2.7 (0.51)	2.7 (0.50)	2.7 (0.49)	NS
***Commitment to organization*** (3) b	7.3 (1.99)	7.3 (1.97)	7.0 (1.99)	7.8 (1.58)	7.4 (1.84)	NS
***Work motives***						
Intrinsic motivation to work (3) a	8.3 (0.94)	8.2 (1.08)	7.7 (1.12)	7.9 (1.08)	8.1 (1.22)	0.004 (Ph/Be&Ps) 0.030 (Ph/Er&Phy)
Extrinsic motivation to work (3) a	7.6 (1.42)	8.0 (1.13)	7.1 (1.68)	8.2 (1.10)	8.0 (1.18)	0.007 (Ph/Er&Phy) 0.010 (Ph/S&H) 0.000 (Ph/RN) 0.002 (Be&PS/RN)
To have good pay and material benefits.(SI) e	2.4 (0.59)	2.3 (0.62)	2.2 (0.67)	2.5 (0.57)	2.1 (0.76)	0.004 (S&H/RN) 0.004 (Ph/RN)

*Abbreviations*: *NS* not significant, *Be&Ps* Behavioural scientists/Psychologists, *Er&Psy* Ergonomists/Physiotherapist, *Ph* Physicians, *RN* Registered nurses, *S&H* Safety and health engineers

a = a higher score relates to a negative impact, b = a higher score relates to a positive impact, e = a higher score relates to the importance of having good pay and material benefits. Analysed with mean (SD) and compared pairwise with Independent Samples Kruskal-Wallis adjusted by Bonferroni correction for multiple tests

Significant at *p* < 0.05

Ergonomist/physiotherapists and registered nurses reported that they found the work climate to be significantly more competitive compared with other professionals (Table 4). Compared with ergonomists/physiotherapists, registered nurses or behavioural scientists/psychologists and physicians reported to a lower extent that they had to demonstrate their ability and competence to others in order to be assigned to attractive tasks or projects (Table 5). Physicians also reported that they found the motivation to work to be less important compared with other professionals (Table 5). Compared with other professionals, safety and health engineers belonged to a permanent working group less often (Table 4). In general, all professionals reported high values on the subscales in the content area “Organizational culture and climate” (Table 4). The different professionals also reported high and similar values on social interactions and empowering leadership (Table 4).

### Harassment and bullying

A total of 7% (*n* = 34/472) reported that they had noticed someone being subjected to harassment or bullying at the workplace during the previous six months, and some reported that they had seen up to seven people being bullied or subjected to harassment at the workplace during the last six months. 3% (*n* = 14/472) reported that they themselves had been subjected to harassment or bullying at the workplace during the last six months.

### Internal consistency

Internal consistency measured with Cronbach’s alpha and mean inter-item correlations had an acceptable range in 15 of the 24 subscales reported in the study. There was one subscale with a Cronbach’s alpha slightly below 0.70 and mean inter-item correlation below 0.40, and eight subscales with a Cronbach’s alpha between 0.27 and 0.65 and a mean inter-item correlation between 0.14 and 0.36.

## Discussion

The results show that the professionals in the OH organization in Sweden have varying experiences of the psychological and social factors at work. Overall, the professionals seem to experience their psychological and social factors at their workplace to be satisfactory. The professionals reported some significant differences in their experiences. The most significant differences are shown in subscales and single items on the “*Job task”* measurement level. On the “*Individual*” measurement level there are similarities but with some significant differences between professionals. There are more similarities than differences between the professionals on the “*Social and organizational*” measurement level. Whether or not the significant differences found in the study have an impact on the clinical setting should be further investigated.

Our interpretation is that the results show that the different professionals have different obstacles to overcome in their work environments. Thus, if improvements in the psychological and social factors in the OH work environment are wanted, then it needs to be recognized that “a one size policy” will be unlikely to work in every case. An exception could be that increased collaboration can be helpful when individuals may learn from and support one another.

Safety and health engineers reported to a significantly lower degree than other professionals that they belong to a permanent working group. This is in accordance with the result of a study in the same population in which the safety and health engineers reported that they have a low degree of partnership within the interprofessional collaboration in the OH context compared with other professionals [13]. Interprofessional collaboration has in other studies proved to be associated with a perception of higher work empowerment and a positive perception of the work environment [22, 23]. Safety and health engineers seem to experience their psychological and social factors at work as having positive effects on the “*Social and organizational*” measurement level even if they do not belong to a permanent working group. The high mean values on the subscales “*support from superiors”*, “*support from coworkers”* and “*social climate”* among all professionals are a reflection of those in that same previously mentioned study in which the respondents reported that the factors of openness, honesty and trust were adequate within the interprofessional team collaboration [13]. The social support from superiors was seen as being important when building a healthy work environment, as were perceived competencies, mastery at work, autonomy and social relationships among employees [24, 25].

A surprising result was that 7% of the respondents reported that they had seen someone at the workplace being subjected to harassment and bullying during the last six months and 3% reported that they themselves had been subjected to harassment and bullying in the same period. This is surprising and remarkable since the OH providers and their employers should be aware of the risk of being exposed to harassment, bullying and victimization at work. This need to be further investigated. The result is comparable to a survey from 2018 where 7–8% of the women and 3–5% of the men reported that their work-related inconvenience/injury was related to harassment and/or bullying from supervisors or co-workers [26]. Health risks that have been associated with social and psychological aspects such as harassment at work include sleep problems and depression [1]. To help prevent the risk of harassment, bullying and victimization a written policy at the work place may be of help [2].

In Sweden it is possible to work within in the OHS without a specialist education in occupational safety and health whereas registered nurses and physicians providing OHS in Finland must be specialists in occupational health care [27]. Since the types and levels of education differs between and within the professionals included in this study, this might have influenced the respondents’ perception of the psychological and social factors in the workplace. In this study we have not distinguished between professionals specialized with advanced education in occupational safety and health such as occupational health nurses and occupational health physicians, and those with first a cycle education in their profession. With less knowledge in safety and health the perception and understanding of psychological and social factors at work might be compromised. The expectations and responsibilities related to the roles of the professionals may then be less clear [28].

The results of the study might have been different if there had been an inclusion criterion requiring that the respondents should have had an advanced degree in occupational safety and health. Besides the educational level, the result may also be explained by the respondents’ occupational health literacy or work health literacy (health literacy: “*entails people´s knowledge*,* motivation and competence to access*,* understand*,* appraise*,* and apply health information in order to make judgments and take decisions in everyday life concerning healthcare*,* disease prevention and health promotion to maintain or improve quality of life during the life course*”) [29, 30]. Experience in years within the profession and aspects of the context may influence the perceived level of work empowerment [22].

The use of self-report questionnaires when measuring workload and experience of social and psychological work environments has been questioned, especially in relation to the objectivity of the measurement [31, 32]. When aiming to describe and compare experiences, self-reported measures are acceptable when trying to gauge subjective experiences. Employees´ psychological wellbeing is associated with health literacy and organizational health literacy (“*an organization-wide effort to transform organization and delivery of care and service to take care of their health*” [33]) and supporting leadership [34]. The results show that the experiences of the social and psychological work environments among the respondents were considered to be satisfactory. Whilst our results seem clear, they must be interpreted with caution since the response rate is low and reasons for not responding or participating might include various contributing factors such as high workloads, or stressors and strains at work.

QPS_Nordic_ is an extensive instrument. When the instrument was constructed a wide range of factors that could be termed the psychological and social factors at work was included [14]. The instrument could be regarded as three different instruments in the way items are categorized into the three conceptual modules or levels: *job task*, *individua*l together with *social and organizational* [18]. Some of the items have a high Cronbach’s alpha, others a low one, and the inter-item relation is also low among some items which is a weakness in this study and this has been shown in other studies as well [16, 18].

There are difficulties in reaching the professionals working in an OH context since there is no registry and GDPR does not allow employers to reveal employees postal or email addresses [35]. The managers were asked to deliver the questionnaire to their employees in this paper-based study, for which the response rate was calculated to be 24%, and this was judged to be a reasonably good compromise way for reaching the respondents at the time of the data collection. In a Finnish study among professionals in the OHS (OH physicians, OH nurses, OH physiotherapists and OH psychologists) carried out in 2015, the researchers chose to let the professional associations deliver a web-based questionnaire and they had an overall response rate among the professionals of 15% (7–24%) [36]. The response rate on mail-based surveys has declined from 77% since the 1970s and is continuing to decline and it is expected to reach as low as 21% in the 2030s [37]. The fact that the response rate is declining may help explain the low response rate in this study. It is problematic that the possibility for reaching out to professionals in the OH context is limited, and it obstructs the possibilities for undertaking research within the OH context [13, 27, 36]. There are few professionals working in the OHS compared with other healthcare settings, but their knowledge and work do have an impact on workers’ health, public health and health literacy and they are often mentioned in work environment research [30]. Therefore, it is important to do research that can further develop and enhance the OHS.

This study contribute valuable knowledge about the perception about the psychological and social work environments among professionals in the Swedish OHS context.

### Limitations

Limitations in this study have been identified and should be noted. Some potential respondents may have chosen not to participate because the study, as noted earlier, was part of a rather extensive survey and this may have led to a selection bias. There was no sample size calculation. The anonymity of the study and the way the survey was distributed did not allow for the possibility of sending out reminders and so it was not possible to know how many people the survey did actually reach. Therefore, reporting of response rate is problematic. It is also possible that selection bias may have occurred when the managers selected the respondents. Research about occupational health professionals in general and about their own work environments in particular is sparse, so therefore the ability to compare results with other studies is limited.

## Conclusion

The present findings indicates that different OH professionals experience their psychological and social factors at work in different ways. Overall, their experiences are mostly satisfactory even though all professionals perceive obstacles in the workplace psychological and social environments in various ways. The main differences are related to the job task measurement level. On the social and organizational level, the experiences between the OH professionals are similar and positive. The respondents’ supervisors and organizations seem to “practice what they preach”. The risk of being subjected to harassment and bullying seem to be the same when working in the OH context as when working in other contexts. This is something that the OH professionals should try to address.

## Data Availability

The data underlying this article cannot be shared publicly due Swedish laws on research ethics and the ethical approval provided by the Swedish Ethical Review Authority.
